# Transcriptional interaction-assisted identification of dynamic nucleosome positioning

**DOI:** 10.1186/1471-2105-10-S1-S31

**Published:** 2009-01-30

**Authors:** Zhiming Dai, Xianhua Dai, Qian Xiang, Jihua Feng, Yangyang Deng, Jiang Wang, Caisheng He

**Affiliations:** 1 Electronic Department, Sun Yat-Sen University, Guangzhou, 510275, PR China

## Abstract

**Background:**

Nucleosomes regulate DNA accessibility and therefore play a central role in transcription control. Computational methods have been developed to predict static nucleosome positions from DNA sequences, but nucleosomes are dynamic in vivo.

**Results:**

Motivated by our observation that transcriptional interaction is discriminative information for nucleosome occupancy, we developed a novel computational approach to identify dynamic nucleosome positions at promoters by combining transcriptional interaction and genomic sequence information. Our approach successfully identified experimentally determined nucleosome positioning dynamics available in three cellular conditions, and significantly improved the prediction accuracy which is based on sequence information alone. We then applied our approach to various cellular conditions and established a comprehensive landscape of dynamic nucleosome positioning in yeast.

**Conclusion:**

Analysis of this landscape revealed that the majority of nucleosome positions are maintained during most conditions. However, nucleosome occupancy at most promoters fluctuates with the corresponding gene expression level and is reduced specifically at the phase of peak expression. Further investigation into properties of nucleosome occupancy identified two gene groups associated with distinct modes of nucleosome modulation. Our results suggest that both the intrinsic sequence and regulatory proteins modulate nucleosomes in an altered manner.

## Background

Nucleosomes are the fundamental repeated units of eukaryotic genomes [1]. They are comprised of 147-bp segments of DNA wrapped around an octamer of histone proteins [2]. The positions of nucleosomes play important roles in diverse cellular processes that rely on access to genomic DNA, including DNA replication, recombination, repair, transcription, chromosome segregation, and cell division [3]. In general, there are three main ways in which cells regulate nucleosomal influences on these cellular processes: chromatin remodeling [4], histone modification [5], and incorporation of histone variants [6]. Recently, high-resolution nucleosome positions across genomes have been identified in yeast (Saccharomyces cerevisiae) [7-11] and human [12-14]. These valuable data make it possible to understand how nucleosome positions are exactly determined in vivo.

The coordination of nucleosome positions is a complex process involving combined interactions among multiple factors. Experimental evidence indicates that certain DNA sequences have strong ability to wrap around the histone octamer [15]. Consequently, the intrinsic DNA sequence is one dominant factor for governing nucleosome positioning. Recent studies have used DNA sequence features to predict genome-wide nucleosome positions with modest success [16-19], confirming that nucleosome positioning is partially encoded in the genomic DNA sequence. On the other hand, other factors also contribute to nucleosome positioning [9,19,20]. One genomic study has shown that the chromatin remodeling complex Isw2 can override the underlying DNA sequence to reposition nucleosomes [9].

It has become clear that nucleosome positions are highly dynamic [21-23]. Recent genome-wide studies have further supported this concept [10,13,24]. Hogan et al. have reported cell cycle-specified fluctuation of nucleosome occupancy at gene promoters [24]. Shivaswamy et al. have identified changes in individual nucleosome positions before and after subjecting cells to heat shock [10]. These studies have also collectively revealed that the dynamic nucleosomal template influences the capacity of genes to alter expression levels in response to various signals. Insights into nucleosome positioning dynamics should enhance our understanding of the mechanism of gene expression. However, as high-resolution measurement of global nucleosome positions is still experimentally costly, there lacks a comprehensive map of dynamic nucleosome positioning in various cellular conditions.

Previous computational methods have predicted static nucleosome positions using DNA sequences with nucleosome formation or inhibition signals [16-19]. However, more information besides the intrinsic DNA sequence is required to model nucleosome positioning dynamics. To our knowledge, there has been no report on computational identification of dynamic nucleosome positions. In this paper, we report a novel computational approach for identifying dynamic nucleosome positioning at gene promoters on the base of dynamic transcriptional interaction and genomic sequence information. Our predictions are in good agreement with experimentally determined nucleosome occupancy available in three cellular conditions. We use our method to offer a landscape of yeast nucleosome positions in various cellular conditions. Insights into this landscape show that nucleosome occupancy at most promoters is negatively correlated with the corresponding gene expression level. The underlying DNA sequence itself tends to account for nucleosome positioning for promoters whose nucleosome occupancy does not fluctuate with their corresponding expression levels. We also find additional features of the global nucleosomal landscape.

## Results and discussion

### Transcriptional interaction is discriminative information for nucleosome occupancy

A recent study has used nucleosome occupancy information to assist identification of transcription factor (TF) binding sites [25]. Conversely, we asked whether TF binding profiles can be used to discriminate nucleosome occupancy profiles. We used yeast data in YPD medium to address this question. We first used k-means clustering to assign 5,446 yeast genes to 50 patterns based on their TF binding profiles (Methods). We next calculated average nucleosome occupancy profile at promoters for each gene cluster, and then computed pair-wise Euclidean distances among these average profiles. The resulting distance reflected the degree of difference between the nucleosome occupancy profiles of two gene clusters. Fixing the number of genes in each cluster, we reassigned 5,446 genes to 50 patterns at random and repeated the calculation of pair-wise Euclidean distances. The average pair-wise distance for genes clustered based on TF binding profiles was greater than any one in 10,000 random experiments (*P *< 10^-27^, Mann-Whitney U-test). Nucleosome occupancy profiles could be well discriminated by the information of TF binding, an important type of transcriptional interactions. This result demonstrates that transcriptional interaction is discriminative information for nucleosome occupancy.

### A novel computational approach for identifying dynamic nucleosome positioning

Motivated by the observation above, we asked whether it is possible to employ TF binding information to predict nucleosome positioning. Indeed, the positions of TF binding sites are strongly associated with nucleosome positions [8,12]. Nucleosomes in promoter regions limit accessibility of DNA to TFs [26], thus TF binding sites typically locate in nucleosome-free regions [7]. Previous studies have indicated that nucleosomes help TFs appropriately bind their targets by exposing functional binding sites and covering those nonfunctional [11,18]. It has also been shown that dynamic regulation of nucleosome positioning is linked to changes in accessibility of DNA to TFs [13]. All the prominent stress-related TFs show a strong increase in accessibility of their binding sites after heat shock, whereas nucleosomes appear to cover nonfunctional binding sites upon transcriptional perturbation [10]. Based on these results, we reason that functional DNA motifs that are associated with TFs tend to be depleted of nucleosomes, while nonfunctional motifs tend to locate into nucleosomes to prevent improper TF binding.

It is well accepted that TFs bind their targets in a dynamic manner, and their corresponding nucleosomal templates undergo dynamic changes [23]. However, dynamic TF binding data during multiple cellular conditions is still unavailable. A question arises concerning how to model this dynamic process. We can identify TFs that can potentially bind their targets at one phase by determining their presence or absence in the cell from their concentration, and can determine their functional and nonfunctional motifs through statistical methods. From a dynamic perspective, if one TF is present at one phase under one cellular condition, its functional DNA motifs tend to be depleted of nucleosomes at that phase, whereas its nonfunctional motifs tend to be covered by nucleosomes then. As TFs differ in their phases of presence, positions of all functional and nonfunctional motifs at one promoter may vary with phases. These differences among phases are linked to changes in nucleosome positions.

**Figure 1:**
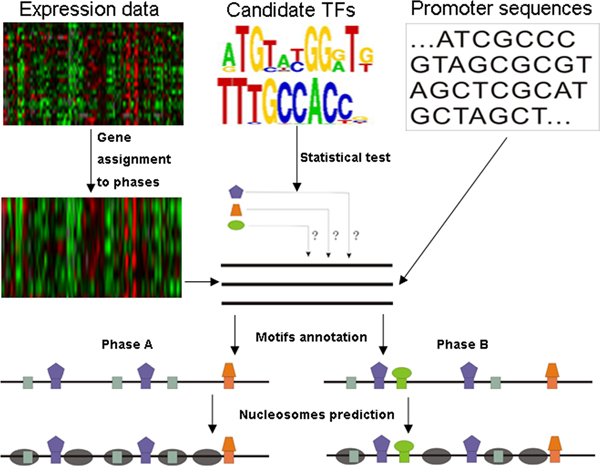
**Overview of the approach**. The procedure takes as input gene expression data during one cellular condition, known position weight matrixes (PWMs) that correspond to candidate TFs and promoter sequences. The method determines phases of presence for each present TF, and combines their binding information with genomic sequence information to identify dynamic nucleosome positions during the condition. Dark sea green squares represent nonfunctional DNA motifs (i.e. motifs unbound by the corresponding present but nonfunctional TFs), while other squares represent functional DNA motifs (i.e. motifs bound by the corresponding present and functional TFs). Gray ellipses represent nucleosomes. Green ellipses, purple pentagons and orange trapezia represent functional TFs.

Based on dynamic transcriptional interaction and genomic sequence information, we developed a novel computational approach for identifying dynamic nucleosome positioning at promoters (Figure 1; Methods). Given gene expression data during one cellular condition, DNA sequences at gene promoters, and known position weight matrixes (PWMs) that correspond to candidate TFs, we could identify nucleosome positioning dynamics during the condition. Using the proposed computational method, we are able to identify dynamic nucleosome positioning during multiple cellular conditions. Since our method only requires gene expression data, known PWMs, and promoter sequences as prior information, it can be widely applied in many organisms. As gene expression data and known PWMs are abundant in yeast, we focus on yeast cellular conditions in this paper.

### Validation of our method

**Figure 2:**
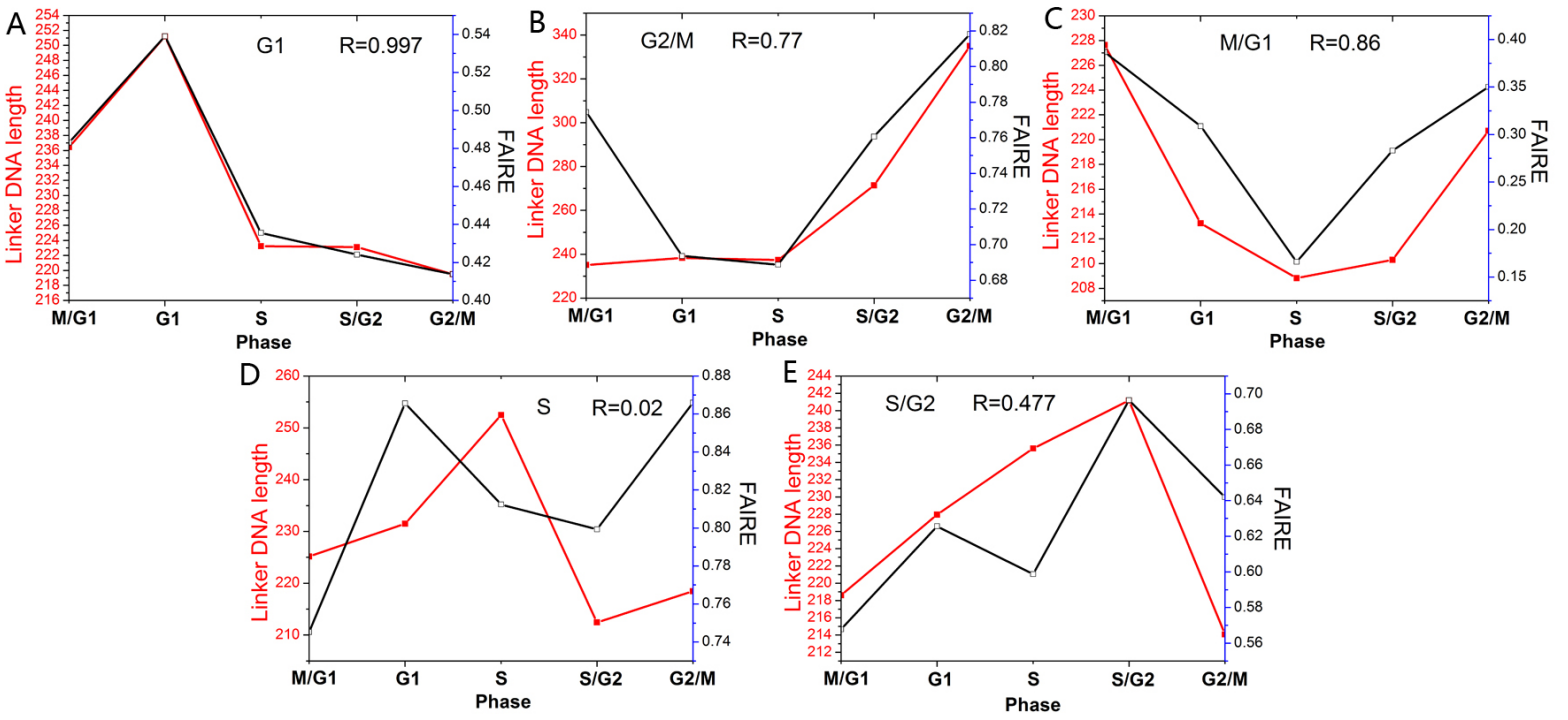
**Correlation between our predictions and experimental measured nucleosome occupancy during cell cycle**. (A) Average enrichment for nucleosome-free regions obtained through FAIRE (black) [24] and average linker DNA length predicted by our method (red) are shown for G1 promoters during cell cycle. R refers to Pearson correlation coefficient between the two profiles. (B) Same as (A), but for G2/M promoters. (C) Same as (A), but for M/G1 promoters. (D) Same as (A), but for S promoters. (E) Same as (A), but for S/G2 promoters.

**Figure 3:**
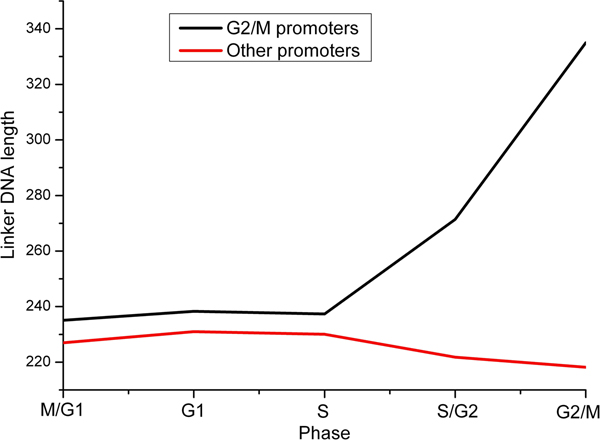
**Comparison of predicted nucleosome occupancy at G2/M promoters and other promoters during cell cycle**. The black line indicates the average linker DNA length of G2/M promoters at each phase throughout the cell cycle. The average linker DNA length of other promoters (red) is also shown.

We first applied our method to cell cycle-regulated genes. Yeast cell cycle-regulated genes have been identified and grouped into five phases during which they display peak expression: M/G1, G1, S, S/G2, and G2/M [27]. We directly applied our method to these classified gene groups. Hogan et al. have used a method termed FAIRE (formaldehyde-assisted isolation of regulatory elements), coupled with whole-genome DNA microarrays, to measure nucleosome occupancy through the yeast cell cycle [24]. The FAIRE enrichment values reflect the enrichment for nucleosome-free regions. The measurements in their experiment are single promoter-resolution, whereas our predictions are individual nucleosome-resolution. For comparison, we used the total length of linker DNA between predicted adjacent nucleosomes to represent the enrichment for nucleosome-free regions at the promoter. As our predictions and experimental measurements have different scales, we assessed our method by comparing their change trends throughout the cell cycle (Figure 2). Our predictions showed good agreement with experimental measurements for G1, G2/M, and M/G1 promoters, and modest agreement for S/G2 promoters. During S phase, nucleosomes are disrupted as the replication fork proceeds and new nucleosomes are deposited onto replicated DNA [28]. As mentioned in original literature [24], changes in nucleosome occupancy during this process may obscure experimental measurement. Our method using transcriptional interaction may not account for the nucleosome occupancy in this complex process. In other words, the disagreement in S promoters is attributable to inaccuracies both in our method and in experimental measurement. Hogan et al. have observed that G2/M promoters are relatively depleted of nucleosomes throughout the cell cycle [24]. Our predictions also reflected this property (Figure 3), suggesting that this phenomenon may be correlated with the distribution of functional motifs at G2/M promoters because our approach is based on TF binding.

To examine whether our approach is applicable to exogenous conditions, we identified nucleosome positioning in the response of cells to hydrogen peroxide [29]. Pokholok et al. have profiled histone H3 occupancy across the yeast genome with an average probe density of 266 bp after subjecting cells to hydrogen peroxide for 20 minutes [30]. We referred to H3 occupancy as nucleosome occupancy, although histone H3 variants are assembled into some nucleosomes [31]. We compared our predictions to experimentally measured H3 occupancy [30]. Predicted nucleosomes had significantly higher H3 occupancy than predicted nucleosome-free regions (*P *< 0.003, Mann-Whitney U-test). We asked whether transcriptional interaction information contributes to the significant correspondence between our predictions and experimental measurements. To this end, we predicted nucleosome positions using only genomic sequence information (Methods). Predicted nucleosomes still had higher H3 occupancy than predicted nucleosome-free regions (*P *< 0.02, Mann-Whitney U-test), but the statistical significance became less. This result validates that transcriptional interaction is important information for nucleosome positioning.

Recently, Shivaswamy et al. have measured genome-wide nucleosome positions after subjecting cells to heat shock for 15 minutes [10]. We applied our method to expression data measured in a similar experimental condition [29]. We evaluated our method by calculating the overlap between experimentally measured and predicted nucleosome positions in base-pair resolution. That is, if our predicted state (covered by nucleosome or nucleosome free) of one base pair is the same as its experimentally measured state, the base pair is considered to be accurately predicted. The result shows that we accurately predicted ~56% of base pairs, compared with ~51% for predictions solely from genomic sequence information. This comparison result reveals that transcriptional interaction information significantly contributes to successful identification of nucleosome positioning.

Taken together, these results validate our method in three available datasets of dynamic nucleosome occupancy. It is noteworthy that both expression data and nucleosome occupancy were not measured in exactly the same experimental medium for either exogenous condition. As expression data is prior information for our method and experimentally measured nucleosome occupancy is used to evaluate our method, these discrepancies in experimental conditions inevitably limit assessment of our method. Nevertheless, our results show that TF binding information improves significantly the performance of prediction, which is based on the intrinsic DNA sequence alone.

### Alandscape of dynamic nucleosome positioning in various conditions

**Figure 4:**
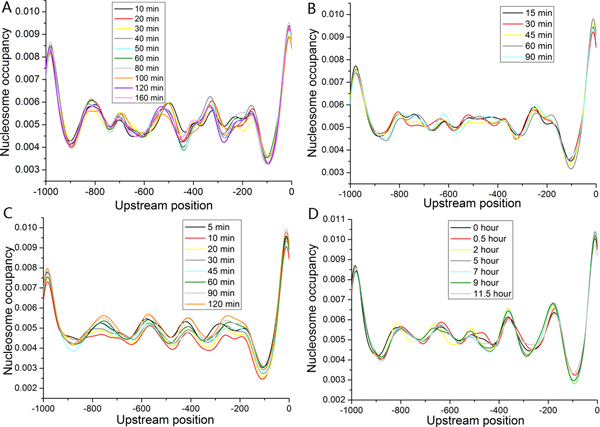
**The maintenance of nucleosome occupancy during selected cellular conditions**. (A) Average identified nucleosome occupancy profiles during condition of hydrogen peroxide are plotted. (B) Same as (A), but for the condition of temperature shift from 37°C to 25°C. (C) Same as (A), but for the condition of DNA damage (ionizing radiation). (D) Same as (A), but for the condition of sporulation.

**Figure 5:**
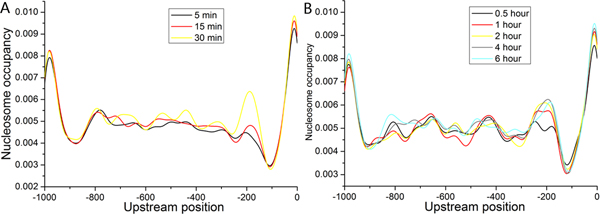
**Drastic changes in nucleosome occupancy during selected cellular conditions**. (A) Average identified nucleosome occupancy profiles during condition of heat shock (from 29°C to 33°C) are plotted. (B) Same as (A), but for the condition of amino acid starvation.

Having validated our approach in available datasets, we applied it to 22 cellular conditions to study global properties of the dynamic nucleosome organization [27,29,32-34]. First, visual inspection of nucleosome occupancy profiles indicated that the majority of nucleosome positions were maintained during most conditions (Figure 4). However, drastic changes in nucleosome occupancy still occurred during some conditions (Figure 5). Investigation into individual nucleosomes revealed that most nucleosome positions were conserved during the corresponding condition: ~66% of positioned nucleosomes were within 30 bp of their positions at the start phases. These results suggest that nucleosome remodeling tends to reposition most nucleosomes on their nearby locations, rather than to give rise to broad region-wide changes. This notion is supported by recent experimental evidence that individual nucleosome positions were largely maintained after heat shock [10].

**Figure 6:**
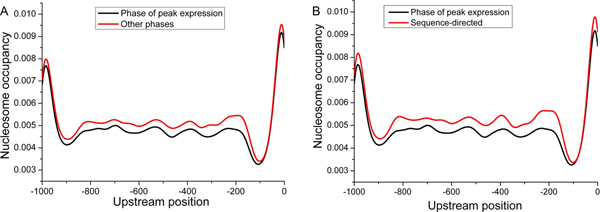
**Comparison of nucleosome occupancy at phases of peak expression and others**. (A) Average identified nucleosome occupancy profiles are plotted for the phases of peak expression (black) and other phases (red) during all conditions. (B) Average identified nucleosome occupancy profiles are plotted for the phases of peak expression (black) during all conditions and the sequence-directed (red).

Second, we studied nucleosome organization by comparing nucleosome occupancy at the phases of peak expression to those at other phases (Figure 6A). Indeed, peak expression was correlated with lower nucleosome occupancy in promoter regions. We asked whether the lower nucleosome occupancy is caused by the intrinsic DNA sequence or other factors. We found that genes also had significantly lower nucleosome occupancy at the phases of peak expression compared to that were predicted based on DNA sequence only (Figure 6B). This result indicates that factors except the intrinsic DNA sequence are associated with the lower nucleosome occupancy. The peak in Figure 6A around -1000 is mainly due to the genomic sequence (see a similar peak in Figure 6B for the nucleosome occupancy predicted based on DNA sequence only).

**Figure 7:**
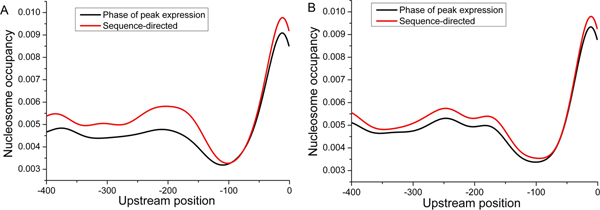
**Nucleosome occupancy of two gene clusters**. (A) Average identified nucleosome occupancy profiles of genes in cluster 1 are plotted for their phases of peak expression (black) and the sequence-directed (red). (B) Same as (A), but for genes in cluster 2.

Third, we further investigated mechanisms of nucleosome positioning. Genes sharing the same phase of peak expression during each cellular condition were clustered into a group. In this way, we obtained a total of 132 groups. Region between -400 and -1 bp upstream of the gene is important for transcription. We focus on nucleosome positioning in this region. Visual comparison between nucleosome occupancy at the phase of peak expression and DNA sequence-directed nucleosome occupancy divided these gene groups into two clusters (Figure 7). Genes in cluster 1 (87 groups) had much lower nucleosome occupancy at the phases of peak expression compared to that DNA sequence-directed. Genes in cluster 2 (45 groups) showed similarity in these two profiles. For genes in cluster 1, DNA sequence-directed nucleosome occupancy cannot ensure transcription for peak expression in the corresponding condition. Some factors (e.g. chromatin remodeling complexes) should override DNA sequences to reposition nucleosomes, paving the way for transcription. For genes in cluster 2, DNA sequence-directed nucleosome occupancy can suffice transcription for peak expression in the corresponding condition.

We next sought to understand mechanisms of nucleosome positioning in other conditions for genes in cluster 2. As microarray experiments for all cellular conditions were carried out with the cell cycle as the start point, we analyzed gene transcription frequency over the cell cycle [35]. Genes in cluster 2 exhibited lower transcription activity than the rest of the genes (*P *< 10^-4^, Mann-Whitney U-test), whereas genes in cluster 1 did not show this property (mean transcription frequency = 7.39 for genes in cluster 1 and 7.42 for the rest of the genes). We hypothesize that the underlying DNA sequence plays less important roles in nucleosome positioning for genes in cluster 2, as their DNA sequence-directed nucleosome occupancy can suffice transcription, which is contradictive with their low transcription activity. To test this hypothesis, we compared experimentally measured nucleosome occupancy profiles to DNA sequence-directed nucleosome profiles in promoter regions [8]. Genes in cluster 2 had lower Pearson correlation coefficients between these two profiles than the rest of the genes (*P *< 10^-5^, Mann-Whitney U-test). This result demonstrates that the intrinsic DNA sequence explains less nucleosome positioning in promoter regions of gene cluster 2. In other words, regulatory proteins should account for more nucleosome positions in promoter regions of gene cluster 2 over the cell cycle. We further analyzed gene activity in various conditions for gene cluster 2. We compiled gene expression data from 1,082 published microarray experiments under various cellular conditions (Methods). For each gene, we calculated the proportion of experiments in which it displayed significantly up-regulated expression changes, and defined the normalized resulting value as open rate. The open rate reflected the general gene activity in various conditions. Genes in cluster 2 showed lower open rates than the rest of the genes (*P *< 10^-6^, Mann-Whitney U-test). Overall, as DNA sequence-directed nucleosome occupancy at gene promoters of cluster 2 may permit accessibility to TFs, regulatory proteins should reposition nucleosomes to prevent improper transcription in repressed state. In contrast, as DNA sequence-directed nucleosome occupancy at gene promoters of cluster 1 can not suffice transcription for peak expression, nucleosome remodeling is required for transcription.

**Figure 8:**
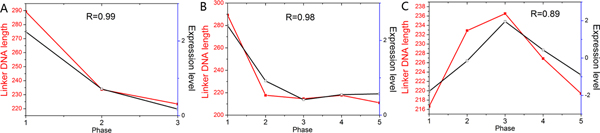
**Correlation between linker DNA lengths and gene expression levels for selected gene groups**. (A) Correlation between average linker DNA lengths (red) and average gene expression levels [29] (black) is shown for genes displaying peak expression at the first phase of heat shock (from 29°C with 1 M sorbitol to 33°C without sorbitol). R refers to Pearson correlation coefficient between the two profiles. (B) Same as (A), but for genes displaying peak expression at the first phase of amino acid starvation [29]. (C) Same as (A), but for genes displaying peak expression at the third phase of heat shock (from 25°C to 37°C) [29].

Finally, we sought to explore the relationship between nucleosome occupancy and gene expression level. As mentioned above, we used total length of linker DNA to represent the enrichment for nucleosome-free regions at the promoter. 88 of 132 gene groups showed high positive correlation (correlation coefficient, *R *> 0.5) between linker DNA lengths and gene expression levels during the corresponding condition (Figure 8). This result is consistent with a general observation that the level of nucleosome occupancy is inversely proportional to the transcription initiation rate at the promoter [36]. But what lead to the low positive correlation for the other 44 groups? One possibility is the retention of nucleosome occupancy throughout the corresponding condition; another one is the inverse changing trend between length of linker DNA and gene expression level. Only 4 of these 44 groups had correlation coefficients less than -0.5. To test the former possibility, we calculated the standard deviation of linker DNA lengths during the corresponding condition for each group. These 44 groups showed lower standard deviation than the other groups (*P *< 10^-3^, Mann-Whitney U-test), indicating that their nucleosome occupancy in promoter regions is relatively maintained during the corresponding condition. Furthermore, these 44 groups showed a moderate overlap with gene cluster 2 (*P *< 0.05, hypergeometric), implying that their maintenance of nucleosome occupancy is linked with the intrinsic DNA sequence.

To sum up, we provided a map of nucleosome positioning in various cellular conditions and provided insights into global characteristics of this map. Moreover, we classified genes based on properties of their nucleosome occupancy, and found that these gene groups are correlated with distinct modes of nucleosome modulation.

## Conclusion

We have developed a novel computational approach for identifying dynamic nucleosome positioning at promoters during cellular conditions, and have successfully predicted the experimentally determined nucleosome positions using this approach. These results demonstrate that the simplified assumptions in our approach are feasible. A combination of transcriptional interaction and genomic sequence information can give good modelling of in vivo nucleosome positioning dynamics. Application of our method established a comprehensive map of dynamic nucleosome positioning during various conditions. Analysis of our predicted nucleosomes revealed mechanisms of nucleosome positioning in various conditions.

Our identifications, based on TF binding and genomic sequence information, showed stronger correspondence with in vivo data than predictions on the base of genomic sequence information alone. This result suggests that TF binding is critical information for nucleosome positioning. However, whether the changes in nucleosome occupancy facilitate TF binding or occur as a consequence of TF binding is not known. TFs Abf1, Cbf1 and Rap1 are involved in nucleosome remodeling as indicated by Gene Ontology [37]. The 132 gene groups did not show significant enrichment for targets of these three TFs, instead, a modest depletion for their targets was observed (*P *< 0.05, chi-test). Although we cannot rule out the possibility that other nucleosome remodeling-related TFs have not yet been identified, one plausible explanation for this observation is that TF binding is not the main source of nucleosome remodeling. We speculate that the main manner of nucleosome remodeling is through nonspecific remodeling complexes to permit or impede site-specific access to TFs. Other study has indicated that nucleosome occupancy plays an instructive role in determining TF Leu3 targeting [38]. A recent study has demonstrated that the chromatin remodeling complex Isw2 repositions nucleosomes to prevent transcription initiation from spurious sites [9]. The studies above have collectively implied that changes in nucleosome occupancy control TF binding. This causal relationship indicates that TF binding also reversely reflect information of nucleosome occupancy. Therefore, we do not only predict nucleosome positioning using its determinant (i.e. genomic sequence) like previous approaches, but also infer nucleosome positioning using its outcome (i.e. TF binding). Our results validated that the incorporation of TF binding information can improve the identification of dynamic nucleosome positioning.

We found that the global characteristics of nucleosome occupancy landscape persist throughout most conditions. Experimental evidence has supported our observation in two conditions (i.e. heat shock and cell cycle) [10,24]. The maintenance of nucleosome organization may be due to three reasons. First, the intrinsic DNA sequence provides a concrete framework for positioning nucleosomes. Nucleosome regulation is implemented upon this framework. Second, nucleosome remodeling is energy cost, and it may not be the most dominant determinant of nucleosome positioning in that yeast cells are likely to be nutrient-limited in their natural environment. One example is that Isw2 influences nucleosome positions of only ~7% of yeast genes [9]. Moreover, nucleosome remodeling does not always result in drastic changes in nucleosome occupancy. Third, nucleosomes should be required to cover spurious motifs to prevent inapposite transcription. DNA motifs of TFs are usually short and degenerate. As a result, there are redundant motifs in the genome. Furthermore, highly degenerate motifs bound by TFs can also contribute to gene expression [39]. Nucleosomes are therefore positioned in a stereotypical manner to protect nonfunctional motifs.

A key finding of this study is that genes exhibit distinct modes of nucleosome modulation. Nucleosome positions are determined by a combination of DNA sequence composition and regulatory proteins. However, it is unclear how these factors work in concert. For genes whose DNA sequence-directed nucleosome occupancy can suffice transcription, regulatory proteins are required to remodel nucleosomes in repressed state. On the other hand, regulatory proteins remodel nucleosomes to activate transcription for genes whose DNA sequence-directed nucleosome occupancy does not enable transcription. We found that these two gene clusters are not cellular condition-specific, and there is no significant difference in DNA sequence preferences for nucleosomes between these two clusters (data not shown). We speculate that these two modes of nucleosome modulation are linked with evolutionarily conservation in nuclear organization and sequence composition. Genes in the former cluster display lower transcription activity over the cell cycle, implying that regulatory proteins are involved in nucleosome positioning for these genes. The interaction between regulatory proteins and nucleosomes should be relatively stable. Regulatory proteins and their targets should be adjacent in nucleus. Otherwise the interaction between them may be transient. For genes in the latter cluster, DNA sequence-directed nucleosome occupancy does not enable transcription, TF binding sites thus should be enriched in regions with high DNA sequence preferences for nucleosomes. Nevertheless, the cause for these two modes of nucleosome modulation remains to be explored.

We have only begun to explore the potential of the application of factors besides genomic sequence information to predict nucleosome positions. Despite the successes described above, our approach still has limitations. Nucleosomes govern the access of DNA to transcription apparatus. However, known TFs represent most, but not all, transcription apparatus. This discrepancy may limit the performance of our approach. As discussed above, TF binding seems to be a consequence of the changes in nucleosome occupancy. Although TF binding is critical information for nucleosome positioning, ATP-dependent chromatin remodeling and histone modification are the main ways of nucleosome remodeling. But they still lack comprehensive experimental data in multiple conditions. Until recently, models for dynamic nucleosome modification have been developed [40,41], which provides the possibility of predicting nucleosome modification. Future studies integrating more information are essential to our understanding of dynamic nucleosome positioning.

## Methods

### Data preparation

Yeast genome sequences were downloaded from the Saccharomyces Genome Database [42]. The TF-binding data set is from Harbison et al. [43], which includes the TF-binding levels of 203 TFs to 5,446 promoters in YPD medium. We used the matrix (with promoters as row entries and with TFs as column entries), with binding ratio as its element, as input for k-means clustering. We used the kmeans function in Matlab with default setting to divide these promoters into 50 groups. Genome-wide nucleosome occupancy data with 4-bp resolution in YPD medium is from Lee et al. [8]. For analysis, we converted the data into 1-bp resolution by linear interpolation. In this way, the nucleosome occupancy profile for each gene between -1,000 and -1 (relative to the +1 ATG translational start codon) was obtained.

We compiled available gene expression data from the Saccharomyces Genome Database [42], a total of 1,082 published microarray experiments for 6,260 genes in various cellular conditions. For each gene, we calculated the proportion of experiments in which it displayed significantly up-regulated expression changes, and defined the normalized resulting value as open rate. To avoid confusion due to experimental noise, we set a relatively strict threshold (2.5-fold) for significantly up-regulated expression changes.

### The proposed computational approach for identifying dynamic nucleosome positions

Given gene expression data during one cellular condition, DNA sequences at gene promoters, and known PWMs that correspond to candidate TFs, we used following procedures to identify nucleosome positioning dynamics during the condition. First, we identified genes displaying significantly up-regulated changes in gene expression (hereinafter referred to as condition-regulated genes) and assigned them to phases (time points for exogenous conditions) at which they display peak expression (hereinafter referred to as phase-related genes), for example, G2/M-phase genes of cell cycle. To identify nucleosome positions for more genes, we set a less strict threshold (2-fold) for significantly up-regulated changes in gene expression. Genes displaying peak expression at the same phase during one condition should tend to be regulated by similar TFs, for example, TFs Mcm1 and Fkh2 regulate G2/M-phase genes [44]. The gene assignment can assist identification of functional and nonfunctional TFs for each condition-regulated gene.

Second, we used known PWMs and statistical test to derive functional and nonfunctional TFs for each condition-regulated gene. We collected 135 known PWMs that correspond to TFs from MYBS [45], a comprehensive web server integrating ChIP-chip data and phylogenetic footprinting data. For each TF, we scored every subsequence in terms of its PWM, and assigned the highest score in each promoter region to the corresponding gene. We also identified positions of DNA motifs at promoters according to thresholds from MYBS. We then tested whether DNA motifs are bound by the corresponding TFs. If the TF binds a subset of genes, their PWM scores should be different from those of the other genes. To examine whether the TF functions during the condition, we used the Kolmogorov-Smirnov (K-S) statistical test to evaluate the difference in the distribution of PWM scores between the condition-regulated genes and the rest of the genes as well as between the phase-related genes and the rest of the genes. The K-S P value provides the statistical significance of the difference between the two distributions. We set 0.01 to the threshold for P value. In this way, we could derive functional TFs, nonfunctional TFs and positions of their DNA motifs for each condition-regulated gene.

Third, we determined the presence or absence of each TF and the phases of presence for each present TF. We can identify TFs that can potentially bind their targets at one phase by determining their presence or absence in the cell from their concentration. However, there still lacks dynamic protein concentration data during multiple cellular conditions. Previous studies have shown that there is a strong correlation between protein and mRNA levels [46], and have also revealed that mRNA abundance can explain 73% of variance in protein levels [47]. We used mRNA level as a close approximation to protein concentration. Like the method in [48], we determined the presence or absence of each TF by assessing its absolute expression level over the cell cycle and relative expression level during the condition, as microarray experiments for all cellular conditions were carried out with the cell cycle as the start point. Jansen et al. have offered a comprehensive reference set of absolute mRNA expression levels by merging and scaling together from a variety of data sets [49]. From the absolute expression data, we grouped TFs into those showing high (greater than 1.5), medium (less than 1.5 and more than 0.5) or low (less than 0.5) abundance. For each TF, if its encoding gene displayed up-regulated (for TFs showing high, medium or low abundance) or moderate down-regulated changes (for TFs showing high abundance) relative to absolute expression level, the corresponding phases were defined as its phases of presence; otherwise it was determined to be absent. For each present TF, its functional motifs are assumed to be depleted of nucleosomes at the phases when it is present, whereas its nonfunctional motifs are assumed to be covered by nucleosomes at the phases of its presence. In this way, we obtained loci in promoter regions covered by nucleosomes or depleted of nucleosomes for each phase.

Fourth, we integrated transcriptional interaction information obtained above and DNA sequence-directed nucleosome formation potential to identify nucleosome positions at each phase. We divided the promoter into some segments, each of which began with the right end of one nucleosome-depleted locus and ended with the left end of the next nucleosome-depleted locus. The genomic DNA sequence itself is one dominant determinant of nucleosome positioning in vivo. The nucleosome positioning in each segment was identified by DNA sequence-directed nucleosome formation potential and the known nucleosome-covered loci. Lee et al. have integrated most sequence features related to nucleosome positioning to model nucleosome occupancy [8], and we used their predicted nucleosome formation potential in this paper. DNA sequences with high affinity for nucleosomes are preferable for nucleosomes. Therefore, nucleosomes are more likely to locate on favourable DNA sequences. For every possible nucleosome covering the nonfunctional motif, the one with the greatest sequence-directed nucleosome formation potential was identified as nucleosome as long as it did not overlap with any previously determined nucleosome. For other nucleosomes, we determined their positions by iterating over the sequence-directed nucleosome formation potential in decreasing order as long as the new nucleosome did not overlap with any previously determined nucleosome. This iteration proceeded until no more nucleosomes could be laid at the promoter.

### Analysis of our predictions

We applied our approach to the following 22 cellular conditions: cell cycle (1 condition) [27], stress response (17 conditions) [29], diauxic shift (1 condition) [32], DNA damage (2 conditions) [33] and sporulation (1 condition) [34]. For cell cycle and sporulation, we directly applied our method to classified gene groups from original publications. Expression data were downloaded from journals' or papers' web supplements.

The approach for predicting nucleosome positions based on genomic sequence alone is as follows: we determined nucleosome positions by iterating over the sequence-directed nucleosome formation potential in decreasing order as long as the new nucleosome did not overlap with any previously determined nucleosome. This iteration proceeded until no more nucleosomes could be laid at the promoter.

We used a Gaussian kernel to model predicted nucleosome occupancy. The mean of the Gaussian was taken as the centre of the identified nucleosome position under consideration, with the standard deviation set at 25 bp. This threshold is set according to the length (147 bp) of nucleosome so that the modelled nucleosome occupancy of predicted linker DNA approximates to 0.

## Competing interests

The authors declare that they have no competing interests.

## Authors' contributions

ZD and XD designed the study, analyzed the results and drafted the manuscript, and ZD also implemented the algorithms, carried out the experiments. QX, JF, YD, JW and CH participated in the analysis and discussion. All authors read and approved the final manuscript.
